# Gene structure, transcripts and calciotropic effects of the PTH family of peptides in *Xenopus *and chicken

**DOI:** 10.1186/1471-2148-10-373

**Published:** 2010-12-01

**Authors:** Pedro LC Pinheiro, João CR Cardoso, Ana S Gomes, Juan Fuentes, Deborah M Power, Adelino VM Canário

**Affiliations:** 1 Centre of Marine Sciences, Comparative Molecular Endocrinology, Universidade do Algarve, Campus de Gambelas, 8005-139 Faro, Portugal

## Abstract

**Background:**

Parathyroid hormone (PTH) and PTH-related peptide (PTHrP) belong to a family of endocrine factors that share a highly conserved N-terminal region (amino acids 1-34) and play key roles in calcium homeostasis, bone formation and skeletal development. Recently, PTH-like peptide (PTH-L) was identified in teleost fish raising questions about the evolution of these proteins. Although PTH and PTHrP have been intensively studied in mammals their function in other vertebrates is poorly documented. Amphibians and birds occupy unique phylogenetic positions, the former at the transition of aquatic to terrestrial life and the latter at the transition to homeothermy. Moreover, both organisms have characteristics indicative of a complex system in calcium regulation. This study investigated PTH family evolution in vertebrates with special emphasis on *Xenopus *and chicken.

**Results:**

The PTH-L gene is present throughout the vertebrates with the exception of placental mammals. Gene structure of PTH and PTH-L seems to be conserved in vertebrates while PTHrP gene structure is divergent and has acquired new exons and alternative promoters. Splice variants of PTHrP and PTH-L are common in *Xenopus *and chicken and transcripts of the former have a widespread tissue distribution, although PTH-L is more restricted. PTH is widely expressed in fish tissue but from *Xenopus *to mammals becomes largely restricted to the parathyroid gland. The N-terminal (1-34) region of PTH, PTHrP and PTH-L in *Xenopus *and chicken share high sequence conservation and the capacity to modify calcium fluxes across epithelia suggesting a conserved role in calcium metabolism possibly via similar receptors.

**Conclusions:**

The parathyroid hormone family contains 3 principal members, PTH, PTHrP and the recently identified PTH-L. In teleosts there are 5 genes which encode PTHrP (2), PTH (2) and PTH-L and in tetrapods there are 3 genes (PTHrP, PTH and PTH-L), the exception is placental mammals which have 2 genes and lack PTH-L. It is hypothesized that genes of the PTH family appeared at approximately the same time during the vertebrate radiation and evolved via gene duplication/deletion events. PTH-L was lost from the genome of eutherian mammals and PTH, which has a paracrine distribution in lower vertebrates, became the product of a specific endocrine tissue in Amphibia, the parathyroid gland. The PTHrP gene organisation diverged and became more complex in vertebrates and retained its widespread tissue distribution which is congruent with its paracrine nature.

## Background

Parathyroid hormone (PTH) and PTH-related peptide (PTHrP) belong to a family of endocrine factors with a highly conserved N-terminal region (amino acids 1-34), which accounts for their overlapping functions in calcium homeostasis [1-3]. In mammals, single copy PTH and PTHrP genes are proposed to share common ancestry [1,3,4] an idea reinforced by the identification of duplicate orthologous genes, PTH1 and 2, and PTHrP1 and 2 in teleost fishes [5-7] which underwent a specific genome duplication [8]. However, the recent identification of a novel PTH-like (PTH-L) gene in teleosts throws into question previous evolutionary models for this gene family [5].

In mammals, PTH is a product of the parathyroid glands and pre-pro-PTH is processed to liberate the biologically active mature 84 amino acid hormone, which regulates serum calcium through its direct actions in bone and kidney counteracting the action of calcitonin [1,9]. In contrast, PTHrP is a pluripotent hormone which acts via intracellular, paracrine and endocrine pathways and regulates cell growth and differentiation, bone development and lactation, and embryonic and fetal development and survival [10-13]. Tissue specific proteolytic processing of PTHrP occurs and generates at least three active fragments [4,11,14], of which only the N-terminal (1-36) fragment has a cognate family 2 G-protein coupled receptor, PTH1R, which also binds PTH [15]. Moreover, alternative promoter utilization and exon splicing generates several different human PTHrP isoforms which range in length from 139-173 amino acids [4,16]. In mammals, a second receptor (PTH2R) is activated by PTH and tuberoinfundibular peptide 39 (TIP39), while in teleost fish PTH2R is only activated by TIP39 [17,18]. Moreover, in teleosts a paralogue of tetrapod PTH1R (designated PTH3R) with affinity for PTHrP also exists [19-23].

PTH and PTHrP have been intensively studied in mammals but their function in other vertebrates is poorly documented. In amphibians, PTH/PTHrP receptors have been characterized and an immunoreactive PTHrP-like peptide with widespread tissue distribution has been detected [24,25]. In chicken, PTH and PTHrP homologues have also been isolated and are involved in chondroblast and osteoblast differentiation, although their role in calcium transport mechanisms is poorly understood [26-31]. Amphibians and birds occupy unique phylogenetic positions, the former at the transition of aquatic to terrestrial life, the latter at the transition to homeothermy, and both organisms have a complex system in calcium regulation. The presence of a parathyroid gland in frogs (not present in fish) coupled with their terrestrial/aquatic environment and the occurrence in birds of a hollow skeleton and heavily calcified eggs are examples of physiological/structural processes which influence calcium and phosphorus requirements.

The identification of new PTH family members in *Xenopus *sp. (Amphibia) and chicken, *Gallus gallus *(Aves) by *in silico *analysis of public databases and gene cloning is reported. Gene structure, gene linkage, alternative splicing and tissue specific transcription are also characterised. The calciotropic activity of *Xenopus *and chicken N-terminal (1-34) peptides of PTH family members *in vitro *are established using *Xenopus *skin and chicken chorionallantois membranes. Finally a model of PTH gene family evolution is proposed and discussed in the context of functional divergence.

## Results

### Xenopus and chicken PTH family members

**Figure 1:**
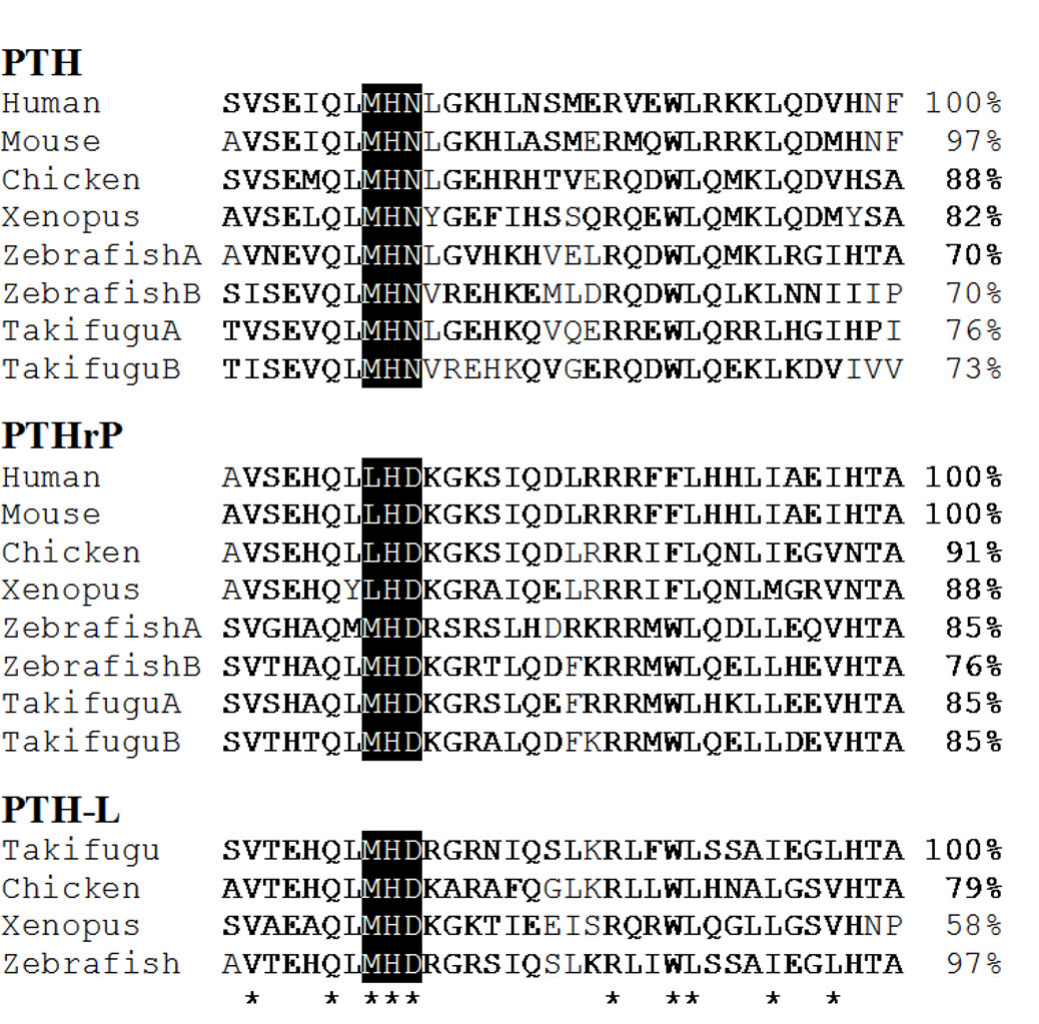
**Multiple sequence alignment of the *Xenopus *and chicken 1-34 PTH family members mature peptide N-terminal region with teleost (*Takifugu *and zebrafish) and mammals (human and mouse). **Conserved amino acid positions identified in all vertebrates are indicated by "*" and percentage of sequence similarity in comparison with human PTH and PTHrP and *Takifugu *PTH-L is given. The typical three amino acid motifs characteristic of each PTH family member in positions 8 to 10 are indicated in black. % similarity to first sequence is indicated on the right. Accession number of the sequences used were: Human (PTH, AAH96144.1; PTHrP, AAA60216); Mouse (PTH, NP_065648; PTHrP, CAC39218.1); Zebrafish (PTHA, NP_998115.1; PTHB, NP_998114.1; PTHrPA, AAY87956.1; PTHrPB, AAY87957.1; PTH-L, CU856139); *Takifugu *(PTHA, CAG26460.1; PTHB, CAG26461.1; PTHrPA, CAB94712.1; PTHrPB, CAG26459.2; PTH-L, CAG26462.1).

Homologues of vertebrate PTH, PTHrP and *Takifugu rubripes *PTH-L genes were identified or deduced *in silico *using the *Xenopus *and chicken genomes (Additional file 1) and validated by cDNA cloning and sequencing (Additional files 2, 3 and 4). The *Xenopus *and chicken PTH-family members shared at least 30% amino acid sequence similarity to the *Takifugu *mature proteins and 58% to the (1-34) N-terminal amino acid residues. Sequence comparison of *Xenopus *and chicken PTH-family members with other vertebrate homologues revealed that specific sequence motifs are conserved (Figure 1 and Additional files 2, 3 and 4). For example, the cleavage sites located before the mature protein in human pre-pro-PTH and PTHrP were conserved. Characteristic proteolytic sites within the human PTHrP protein, which generate three distinct peptides were also conserved: N-terminal PTHrP(1-36), mid-region PTHrP (38-94) and C-terminal PTHrP (107-139) [12] (Additional files 2 and 3). The first 10 N-terminal amino acids, involved in the calcitrophic action of the hormones is the most highly conserved domain. Specific motifs such as, M-H-N in PTH, and L-H-D in tetrapod PTHrP and the amino acids important in receptor-binding in mammals, L^24 ^and L^28 ^are also conserved. Additional conserved residues in *Xenopus *and chicken PTH-family members are V^2^, Q^6^, H^9 ^and R^20 ^**(**Figure 1**)**, suggesting they are important in peptide function from fish to mammals.

### Gene structure and transcript isoforms

**Figure 2:**
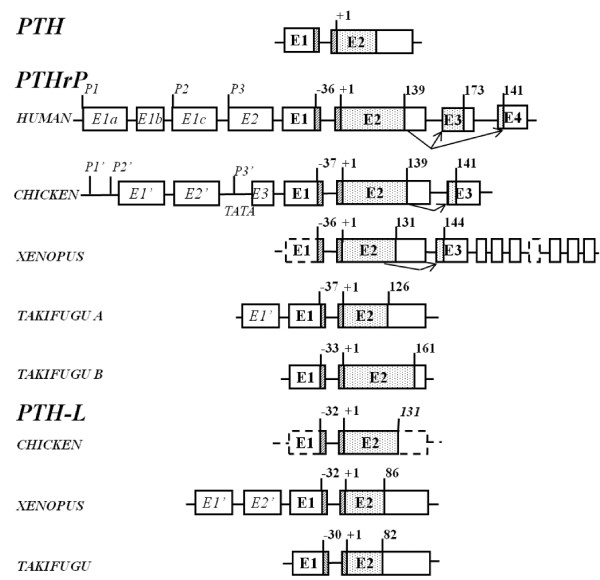
**Gene organization of the vertebrate PTH-like family members.** Exons are represented by boxes and lines indicate introns. Coding (E1 to E4) and non-coding (E1' to E'3) exons are numbered and annotated in bold and italics, respectively. Dotted-filled boxes represent the mature coding regions and black lines box the signal peptide sequence. Arrows represent alternative splice isoforms identified in *Xenopus *and chicken and previously reported in human. The general organization of the conserved vertebrate PTH gene structure is represented and the size of vertebrate PTHrP and PTH-L precursors is given (amino acids). The length of the chicken PTH-L precursor was predicted *in silico *and is indicated in italics. Dashed lines indicate incomplete structures that were not confirmed *in silico *or amplified by RT-PCR. The start of the mature peptide (+1) and the size of the signal peptide for all vertebrate PTH family members is indicated. The localization of the human PTHrP promoter regions (P1, P2 and P3) and the chicken PTHrP putative promoter sites (P1', P2' and P3') and TATA box consensus sequence within the region of P3' are shown. The figure is not drawn to scale and *Takifugu *A structure was taken from Power et al. [42].

Comparison of gene structure indicates that amphibian and chicken PTH and PTH-L gene organization is conserved and that the peptide precursors are encoded by two separate exons (E1 and E2) (Figure 2). In contrast, the gene structure of PTHrP from *Xenopus *and chicken is poorly conserved and 3 exons code for the mature protein. Alternative splicing generates two distinct transcripts that differ in their 3' region and modify the predicted amino acid sequence of the C-terminal domain (Additional file 4).

#### Transcripts - PTH

The *Xenopus *PTH gene was deduced *in silico *and revealed that exon 1 encodes the pre-pro-region of the protein and exon 2 the mature protein (Figure 2). *Xenopus *PTH cDNA was amplified by RT-PCR from the intestine (accession number FM955441) and the predicted protein has 118 amino acids and yields a mature protein of 87 amino acids. It was not possible to establish the nucleotide sequence of the 5' and 3' UTR of *Xenopus *PTH. The gene structure of chicken PTH was identical to the amphibian homologue but no evidence of alternative splicing was found (Figure 2). Chicken PTH has 119 amino acids and is identical to the previously described cDNA [NM_205452, [32,33]]. Two ESTs were identified (CV890868 and CV041147) from cDNA of a mixture of chicken tissues (whole brain, ultimobranchial gland, parathyroid gland, cecum, tonsil, and primordial germ cells) and were identical to full-length PTH (accession numbers NM_205452 and M36522).

#### Transcripts - PTHrP

The deduced *Xenopus *PTHrP gene is composed of at least 10 exons (Figure 2). The first putative exon contains the pre-pro-peptide and part of the 5'UTR, although the full gene structure in this region remains to be elucidated. Exon 2 encodes the mature protein and part of the 3'UTR and yields Xenopus PTHrP(1-131) (XenPTHrP). The largest XenPTHrP transcript identified has 144 amino acids and results from intra-exon splicing of exon 2 to exon 3 as occurs in the homologue region (osteostatin) in the human PTHrP gene. Through alternative splicing, exon 3 donates 32 amino acids to the predicted protein and at least seven downstream exons generate a unique 3'UTR domain. *Xenopus *PTHrP encodes the largest 3'UTR region identified for this gene in vertebrates.

Two previously published mRNA (NM_205338 and AB175678) [34,35] encode XenPTHrP 1-139 and 1-141 which share an identical amino acid sequence with the exception of two extra arginine (R) residues in the latter isoform contributed by exon (E3) (Figure 2). Two ESTs identified in the tailbud (stage 28-30) (accession numbers CR437266; CR433007) encode a predicted XenPTHrP of 180 amino acids which yields a mature protein of 144 amino acids [XenPTHrP(1-144)]. A second XenPTHrP isoform encoding a protein of 167 amino acids which generates a mature peptide of 131 amino acids [XenPTHrP(1-131)] was predicted from analysis of genomic sequences. The existence of XenPTHrP(1-131) transcript was confirmed by RT-PCR of bone (accession number FM955442).

The deduced chicken PTHrP gene is composed of at least 6 exons (Figure 2 and Additional file 5). The first three exons undergo alternative splicing and contain part of the 5'UTR (Additional file 5). Exon 4 encodes the remaining 5'UTR and part of the pre-pro-protein. The mature protein and part of the 3'UTR of chicken PTHrP(1-139) are included in exon 5. In common with human an intra-exon splice of exon 5 to 6 give rise to chicken PTHrP(1-141). This final exon encodes for the last 3 amino acids of PTHrP(1-141) and the 3'UTR.

Several chicken PTHrP (ckPTHrP) isoforms which differed in the 5' UTR and coding region were identified amongst isolated ESTs (Additional file 1). Five ESTs (BU252785, BU384898, BM489067, ENSGALESTT00000030972 and BU252877) differing in their 5'UTR region, encoded a ckPTHrP of 141 amino acids (Additional file 5). No ESTs for putative ckPTHrP(1-139) were identified in database searches although RT-PCR revealed it is expressed in several tissues (Additional file 6).

#### Transcripts - PTH-L

The gene structure of *Xenopus *PTH-L has only been partly elucidated and was composed of at least two exons and appears to have an identical gene structure to PTH. The deduced protein sequence of PTH-L (accession number FM955443) is 152 amino acids (Figure 2).

Two main transcripts for Xenopus PTH-L (XenPTH-L) were identified by clustering seven XenPTH-L ESTs (Additional file 1). One transcript (XenPTH-L*5utrE1'*) was represented by three ESTs from gastrula stages 10.5-12 (AL964863) and a mixture of brain and spinal cord from tadpoles stages 58-64 (CN076481; CN076482). The second transcript (XenPTH-L*5utrE2'*) was represented by four ESTs from gastrula stages 10.5-12 (AL775245; AL965929; BX750398; BX764109). The deduced XenPTH-L was 118 amino acids long and yielded a mature protein of 86 amino acids (Figure 2 and Additional file 2).

The chicken PTH-L gene had a similar structure to the PTH gene and was composed of two exons and one intron. No ESTs for ckPTH-L were identified although RT-PCR indicated it is expressed in several tissues (Additional file 6).

### Phylogenetic analysis

**Figure 3:**
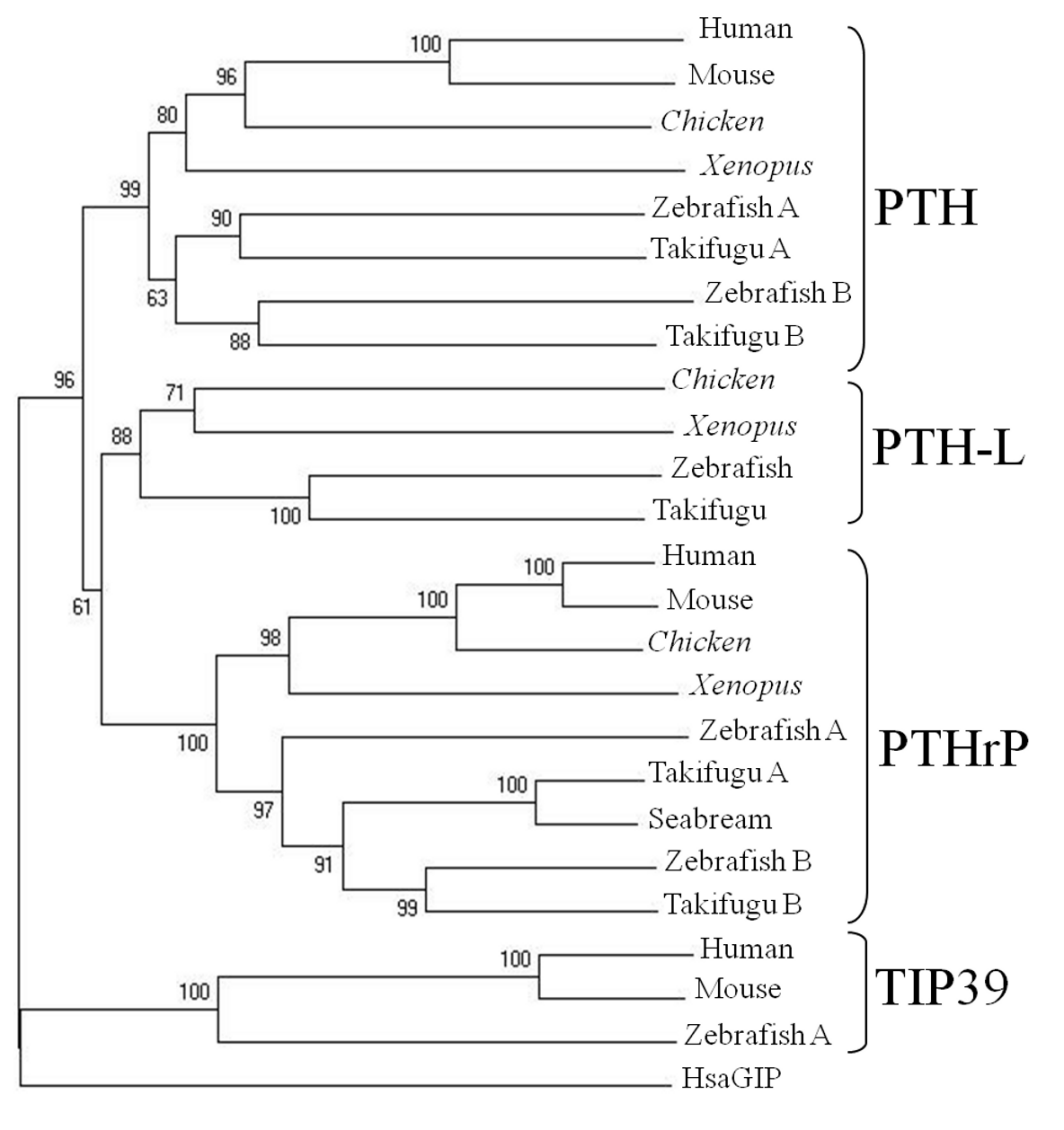
**Consensus phylogenetic tree of *Xenopus *and chicken PTH family members using the Neighbor Joining method **[60]**and 1000 bootstraps replicates with the complete amino acid precursor sequence in Mega3.1 software **[61]**with the settings pairwise deletion, p-distance model and 222 informative sites. ***Xenopus *and chicken PTH family members are in italics and the sequence of human GIP (HsaGIP, NP_004114) was used as outgroup. Human (NP_848544), mouse (NP_444486) and zebrafish (NP_991140) TIP39 mature protein sequences were included for comparative purposes. The accession numbers of other sequences utilized for tree construction are indicated in Figure 1 and seabream PTHrP is AAF79073.

The topologies of phylogenetic trees constructed with Neighbor Joining or parsimony methods were similar and a consensus tree is presented (Figure 3). PTH, PTHrP and PTH-L were separated into different clades and the *Xenopus *and chicken PTH-like family members clustered with their respective vertebrate homologues (Figure 3). In teleosts, a specific duplication of the PTH and PTHrP genes occurred although only one PTH-L exists and this may be a consequence of; 1) deletion of the second copy from teleost genomes or 2) a partial duplication of teleost PTH-like members. The isolation of homologues of *Takifugu *PTH-L gene in amphibian and chicken genomes suggests that members of the PTH/PTHrP family emerged prior to the teleost divergence and were subsequently maintained in vertebrate genomes. The exception is eutherian mammals which lack a PTH-L gene.

### Short-range gene environment comparisons

**Figure 4:**
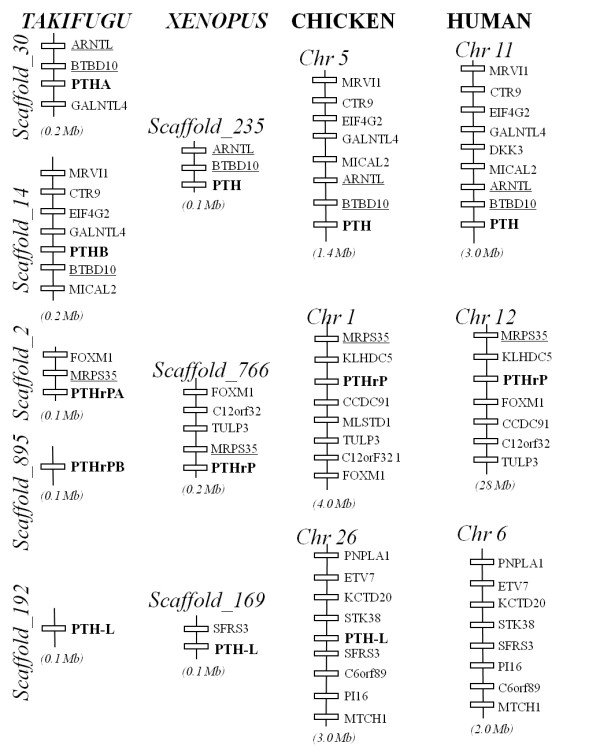
**Short-range gene linkage comparisons of the PTH family members in the *Takifugu*, *Xenopus*, chicken and human genomes. **Genes are represented by closed boxes and the size of the chromosome region analysed is given underneath. Genes were named using HUGO and lines indicate chromosome/scaffold segments. The vertebrate PTH family members are in bold and conserved flanking genes identified within the homologue regions are underlined. The PTH gene is localized in *Xenopus *scaffold_235 and in chicken chromosome 5 and two conserved genes ARNTL and BTBD10 were identified. The *Xenopus *and chicken PTHrP maps to scaffold_766 and chromosome 1, respectively and the gene MRPS35 was found in close proximity in all vertebrate regions analysed. PTH-L and SFRS3 genes map to *Xenopus *scaffold_169 and to chicken chromosome 26. SFRS3 was not linked to *Takifugu *PTH-L and is present on human chromosome 6 which lacks PTH-L. For simplicity, only genes with correspondence across species are represented. The figure is not drawn to scale.

To better understand the evolution of vertebrate PTH family members, the gene environment of the *Xenopus *and chicken genes were characterized and compared with the homologue regions in *Takifugu *and human. Short-range comparisons indicate that gene synteny and gene order were maintained across vertebrates suggesting that members of this family evolved under conservative pressures **(**Figure 4**)**. The chicken and human homologue genome segments were the most highly conserved and similar linked genes were identified flanking PTH-family members. The genes *ARNTL *and *BTBD10 *were localized in close proximity to *PTH*. Genes *MRPS35 *and *SFRS3 *were identified within the *PTHrP *and *PTH-L *homologue regions, respectively. The genes flanking *PTH-L *in *Xenopus *and chicken were identified on human chromosome 6, although PTH-L was lacking suggesting that specific gene/genome rearrangement events occurred during the mammalian radiation.

### PTH family genes expression

**Figure 5:**
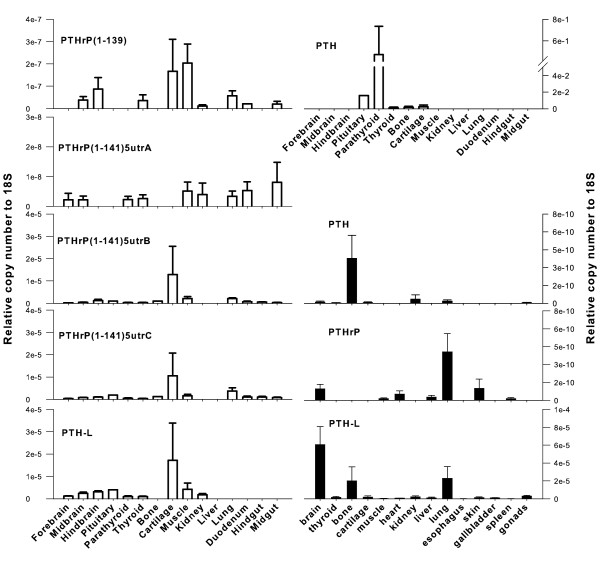
**Expression of chicken (open bars) and *Xenopus *(closed bars) PTH family genes as determined by q-PCR. **Gene specific primers were used to amplify PTH and PTH-L transcripts and PTHrP spliced isoforms from several tissues. The number of amplified transcripts is presented in relation to 18S copy number and data is presented as mean ± S.E. (n = 2 to 3 for *Xenopus *and n = 3 for chicken except for pituitary where n = 1).

The tissue distribution and relative abundance of all *Xenopus *and chicken PTH family transcripts was investigated by RT-PCR (Additional file 6) and q-PCR (Figure 5). PTH and PTHrP transcripts had a similar tissue distribution in both *Xenopus *and chicken.

PTH-L had a widespread tissue distribution in *Xenopus *and the highest transcript abundance (copy number relative to 18S) was detected in brain, lung and bone (Figure 5). *Xenopus *PTHrP transcripts were detected in brain, lung, skin and heart and were low abundance in spleen and muscle (Figure 5). It was not possible to design q-PCR primers which discriminated between the two *Xenopus *PTHrP transcripts. However, RT-PCR (Additional file 6) revealed both transcripts have a similar distribution. *Xenopus *PTH was expressed in bone, kidney, lung and nervous tissue (mixed brain and pituitary). The presence of PTH family members was not established in the amphibian parathyroid gland as its small size and variable morphology made it difficult to collect [36].

In chicken, PTH transcripts were highly abundant in the parathyroid gland (Figure 5), although they were also detected at low abundance in the pituitary, thyroid, bone and cartilage. Chicken PTHrP transcripts were widely expressed (Figure 5) and the various isoforms had a differential tissue distribution suggesting they may have different functional roles. ckPTHrP(1-141)*5utrB *and ckPTHrP(1-141)*5utrC *were the most abundant transcripts (copy number relative to 18S) and were mainly found in cartilage. ckPTHrP(1-141)*5utrB*, ckPTHrP(1-141)*5utrC *and ckPTHrP(1-141)*5utrD *transcripts had a similar tissue distribution. The tissue distribution of ckPTHrP(1-139) overlapped with ckPTHrP(1-141)*5utrA *with the exception of cartilage in which the latter transcript was absent. ckPTHrP(1-139) was highly expressed in muscle and was the only isoform which was not detected in the parathyroid gland. It was also absent from bone together with ckPTHrP(1-141)*5utrA*. In contrast, both isoforms were expressed in kidney where ckPTHrP(1-141)*5utrB *and PTHrP(1-141)*5utrC *were not detected. ckPTH-L transcripts had a similar tissue distribution to ckPTHrP (Figure 5) and were most abundant in cartilage but were also present in pituitary, thyroid, parathyroid glands, muscle, hind brain and kidney.

### Transepithelial calcium transport

**Figure 6:**
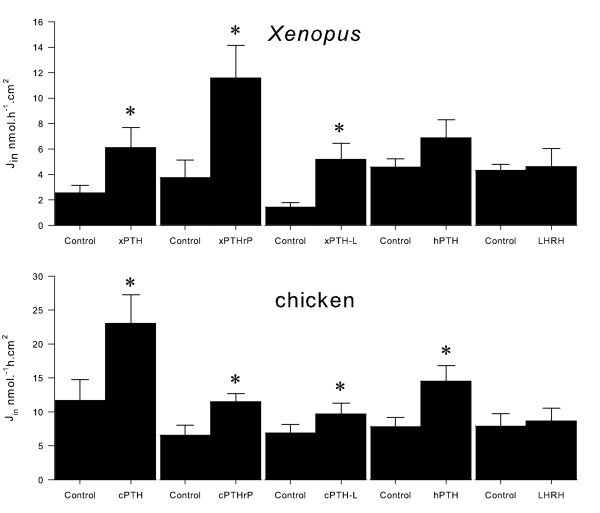
**Calcium fluxes (water to blood side) in *Xenopus *abdominal skin and chicken 16 to 18 day old embryo CAM (shell to embryo side) after the addition of 10 nM, N-terminal (1-34) PTH, PTHrP and PTH-L to the basolateral membrane site. **Human PTH (1-34) and salmon Luteinizing hormone-releasing hormone (LHRH) were used as positive and negative controls respectively. Results are shown as mean ± SEM and the "*" indicates statistical significance compared to control (time 0) (p < 0.05).

The resistance and short-circuit current of *Xenopus *abdominal skin and chicken CAM membranes were substantially different (Table 1). CAM is a leaky epithelium and the frog skin is a tight epithelium and both have calcium transporting capacity. All *Xenopus *and chicken (1-34) N-terminal peptides were able to increase unidirectional calcium fluxes from the apical/basolateral membrane site (Figure 6**)**. XenPTH(1-34) changed calcium fluxes across *Xenopus *abdominal skin from 2.6 nmol.h^-1^.cm^2 ^to 6.1 nmol.h^-1^.cm^2^, XenPTHrP(1-34) from 3.8 nmol.h^-1^.cm^2 ^to 11.6 nmol.h^-1^.cm^2 ^and XenPTH-L(1-34) from 1.4 nmol.h^-1^.cm^2 ^to 5.2 nmol.h^-1^.cm^2 ^. A significant increase in short-circuit current (Isc) was observed after 50 and 60 minutes of exposure of *Xenopus *abdominal skin to XenPTHrP(1-34) and XenPTH-L(1-34), respectively which suggests these peptides may also be involved in the transport of other ions in the skin (Table 1). This possibility was tested by comparing theoretical and measured short-circuit current using the formula *Isc = Ji.z.F *(where *Ji*, represents the uptake, *z *the calcium valence and *F *is the Faraday constant) [37]. For PTHrP the calculated value for *Isc *(in μ/cm^2^) was 0.84 vs. the measured value 0.32. For PTH-L the calculated value for *Isc *was 0.41 vs. the measured value 0.24. The values of *Isc *for the two peptides are within the same range. The difference between calculated and measured values of *Isc *is indicative of an additional transport mechanism responsive to both peptides, e.g. secretion of anions (likely chloride).

**Table 1:** Variation in bioelectric values of Xenopus abdominal skin and chicken CAM measured in vitro prior and at different times after the basolateral application of 10 nM of Xenopus or chicken PTH(1-34) family peptides.

		Time after hormone application (min)						
**Xenopus**		**0**	**10**	**20**	**30**	**40**	**50**	**60**
	
PTH (n = 11)	Isc	2.49 ± 0.34	2.45 ± 0.33	2.43 ± 0.32	2.44 ± 0.30	2.48 ± 0.29	2.48 ± 0.30	2.49 ± 0.28
	Rt	1143 ± 128	1157 ± 129	1164 ± 129	1130 ± 126	1160 ± 127	1164 ± 128	1167 ± 27
PTHrP (n = 12)	Isc	2.59 ± 0.31	2.63 ± 0.30	2.67 ± 0.29	2.74 ± 0.29	2.82 ± 0.28	*2.88 ± 0.27	*2.91 ± 0.25
	Rt	1329 ± 140	1320 ± 122	1328 ± 122	1276 ± 104	1273 ± 98	1263 ± 95	1264 ± 95
PTH-L (n = 12)	Isc	2.36 ± 0.42	2.36 ± 0.43	2.39 ± 0.42	2.44 ± 0.42	2.51 ± 0.42	2.56 ± 0.43	*2.59 ± 0.43
	Rt	1332 ± 81	1328 ± 75	1335 ± 79	1318 ± 79	1327 ± 78	1322 ± 76	1335 ± 76
**Chicken**								
PTH (n = 11)	Isc	7.41 ± 1.90	7.54 ± 1.84	7.39 ± 1.86	7.33 ± 1.88	7.02 ± 2.00	7.14 ± 2.06	7.03 ± 2.08
	Rt	190 ± 20	191 ± 20	194 ± 21	196 ± 21	191 ± 23	194 ± 23	196 ± 24
PTHrP (n = 9)	Isc	12.53 ± 2.77	13.09 ± 2.76	13.02 ± 2.74	13.05 ± 2.73	13.03 ± 2.69	13.22 ± 2.76	13.06 ± 2.73
	Rt	181 ± 15	182 ± 15	181 ± 15	179 ± 15	174 ± 15	176 ± 15	177 ± 16
PTH-L (n = 12)	Isc	7.22 ± 1.26	7.37 ± 1.18	7.15 ± 1.18	6.86 ± 1.10	6.75 ± 1.08	6.80 ± 1.09	6.66 ± 1.10
	Rt	211 ± 21	208 ± 20	211 ± 20	207 ± 21	203 ± 20	205 ± 20	207 ± 30

Note: Isc (μAmp/cm^2^); Rt (Ω.cm^2^); * indicates statistically significant difference from time = 0 control (p < 0.05)

ckPTH(1-34) doubled calcium flux through CAM from 12.5 nmol.h^-1^.cm^2 ^to 23.5 nmol.h^-1^.cm^2^, ckPTHrP(1-34) from 6.5 nmol.h^-1^.cm^2 ^to 11.5 nmol.h^-1^.cm^2 ^and ckPTH-L(1-34) from 6.8 nmol.h^-1^.cm^2 ^to 9.8 nmol.h^-1^.cm^2 ^(Figure 6**)**. Human PTH(1-34) induced a significant increase in transepithelial calcium transport in chicken CAM but had no effect on *Xenopus *abdominal skin (Figure 6**)**. Salmon LHRH was used as a negative control and had no effect on calcium transport in either skin or CAM confirming the specificity of the effects observed with the chicken and *Xenopus *peptides.

## Discussion

In the current study, three PTH family members, PTH, PTHrP and PRH-L, were characterized in *Xenopus *and chicken. Preliminary functional analysis suggests the action on calcium uptake of the (1-34) N-terminal region is conserved in PTH family members. Comparison of sequence, structure and gene environment suggests they have evolved by gene duplication and deletion events. Moreover, a number of novel PTHrP splice variants with a variable 5'UTR were also identified. All three PTH family members are present in teleost and tetrapod genomes with the exception of eutherian mammals which have lost the PTH-L gene, probably because of specific gene or genome rearrangements during the mammalian radiation. This hypothesis is supported by the conservation in human chromosome 6 of genes linked to PTH-L in fish, *Xenopus *and chicken. Phylogenetic analysis supports previous theories that PTH-like family members evolved from a common ancestral precursor, from which tuberoinfundibular peptide 39 (TIP39) has also been suggested to have arisen [38]. To date, PTH family homologues remain to be identified in invertebrates and their emergence in vertebrates coupled with their important role in calcium homeostasis and skeletal development suggest that their origin may be associated with the acquisition of a calcified endoskeleton.

Analysis of PTH family members in *Xenopus *and chicken indicates that similar post-translation modifications to those previously described for the human homologue occur [9,16]. The bioactive N-terminal (1-34) peptide of PTH family members is the most conserved region of the protein (Figure 1). The physicochemical properties of amino acids in the N-terminal region including L^24 ^and L^28 ^which are important for receptor activation [39,40], are maintained in *Xenopus *and chicken suggesting they may have similar functional roles across the vertebrates. Moreover, within this region the highest conservation is found in the first residues. Previous studies comparing activation of teleost and human PTHR by PTHrP and PTH suggests they have distinct activation profiles and indicate the C-terminal region of the protein is also involved [20,23,41].

In addition to amino acid sequence conservation, the gene organization of *Xenopus *and chicken PTH and PTH-L is also maintained (Figure 2) [14,42]. However, although PTH-L splice isoforms have been found in *Xenopus *and *Takifugu *[5] no alternative PTH splice isoforms have been identified in any species to date. In contrast, the gene structure of PTHrP is poorly conserved between vertebrates and unique *Xenopus *and chicken isoforms which result from exon skipping events, were identified and resemble those reported for the human gene [12]. In human, three PTHrP transcripts are expressed as a result of alternative promoter usage, two of which (139 and 141 deduced amino acids) are identical in size to those present in chicken. The only difference between the mature proteins in both human and chicken is the presence in the 1-141 PTHrP isoform of two extra C-terminal amino acids (Additional file 4). In *Xenopus *a unique PTHrP splice isoform results from a splice event within the osteostatin region and the incorporation of a novel coding sequence and 3'UTR giving rise to a longer peptide precursor (144 amino acids). In contrast, to terrestrial vertebrates including *Xenopus *where splice variants of PTH family members are common, none were identified in teleosts. Variation in the UTR has been associated with tissue-specific [43], developmental stage or cell state specific [44] regulatory mechanisms. The existence of splice variants suggests that complex regulatory mechanisms for this gene family were acquired within the tetrapod radiation and this may be related to the change in mineral homeostasis which accompanied the adaptation of vertebrates to a terrestrial environment [3,14,45].

The presence of distinct *Xenopus *and chicken PTHrP isoforms and their widespread distribution and expression in bone, cartilage, skin and kidney (classical tissues involved in calcium regulation) further supports their role as important calciotropic factors. The PTHrP transcripts identified in chicken are predicted to produce mature proteins with the same length as the human PTHrP(1-139) and PTHrP(1-141) proteins, although no homologue of the human PTHrP(1-173) was identified suggesting it may be a human innovation. The *Xenopus *and chicken PTHrP and PTH-L tissue expression overlaps in the majority of the tissues analysed suggesting widespread paracrine actions for the two proteins.

PTH distribution is more restricted and in chicken, as expected, is expressed abundantly and almost exclusively in the parathyroid gland (Figure 5). In *Xenopus*, we were not able to identify the parathyroid tissue and it has previously been noted to be inconspicuous and readily degenerates [36,46]. In chicken PTH expression was also detected in the pituitary gland and in *Xenopus *brain/pituitary extracts, which is in agreement with studies which showed that immunoactive protein was detected in pituitaries from sheep [47], and fish [3,48] (which lack an organized parathyroid gland structure). PTH immunoreactivity in brain and pituitary was also reported for several tetrapods, including chicken [49], but so far has only been confirmed in rabbit and rat brain/pituitary. Taken together these results suggest a high degree of conservation of pituitary PTH expression and possibly secretion across vertebrates, although in mouse the thymus is also a source of PTH [50]. PTH expression in *Xenopus *lung has not, to our knowledge, been previously reported. However, the overall PTH tissue distribution largely coincides with the two PTH/PTHrP receptors identified in this organism [25]. This supports the hypothesis that PTH may have a more paracrine action in lower vertebrates.

Although there was an overlapping tissue distribution of some of the transcripts of the three PTH family members, expression of PTH and PTH-L, unlike PTHrP, is more restricted (Figure 5). The PTHrP isoforms identified in chicken seem to conform to specific patterns and levels of abundance, although only one of the transcripts produces a different protein. With the exception of chicken PTHrP(1-141)*5utrA*, the other chicken PTHrP are expressed in cartilage at high levels confirming their important role in this tissue [51]. However, in *Xenopus *PTHrP transcript seemed to be absent from cartilage. The different tissue expression profiles of PTH family members in *Xenopus *and chicken (and *Takifugu *[5]) suggest that specific modulation of gene expression occurs and detailed analysis of the promoter region may help clarify this question.

All *Xenopus *and chicken PTH-like peptides were found to promote unidirectional calcium influx (Figure 6). The differing effect of human PTH(1-34) in chicken and *Xenopus *is in keeping with what has been previously observed for the bovine peptide [52,53]. However, after pre-treatment with Vitamin D, bovine PTH(1-34) is also able to stimulate calcium transport across the *Rana pipiens *skin epithelium [52]. *Xenopus *PTH-L and chicken PTH peptides were the most effective in promoting calcium transport across the membranes in each specific assay. Similar results were obtained for transport across teleost larvae epithelia [5] and although more studies are required to establish their specific function, the results provide new insight into the interlink between evolution and function of the PTH family.

Although the three peptides enhance calcium transport, only PTHrP and PTH-L caused changes in the short-circuit current in *Xenopus *skin, indicating an additional effect on transport of ions other than calcium (Table 1). Similar effects on the short-circuit current in the same range of PTH concentrations have previously been observed with A6 cells derived from *Xenopus *kidney [54]; on sodium transport of PT cells derived from chicken kidney [55]; and with PTHrP on fish intestine [56]. Our results further substantiate the effect of the PTH family of proteins on the short-circuit current and suggest that the action of the different peptides on the short-circuit current is tissue dependent and may vary with species.

While PTHrP and PTH genes have been identified in teleosts and tetrapods, PTH-L seems to be absent from eutherian mammals and it was recently suggested to be the functional homologue of mammalian PTH, in fish [5]. In the present study, *Xenopus *PTH-L in common with teleost PTH-L, was the most potent peptide promoting calcium transport across epithelia. In chicken, a different scenario was observed and PTH seemed to be more efficient in stimulating calcium transport. Taking into consideration a) the reduced calciotropic activity of PTH-L in chicken, b) the overlapping distribution between PTH-L and PTHrP and c) the absence of a PTH-L in the human genome (data not shown), it is proposed that a transitory functional role between the vertebrate PTH-L and the tetrapod PTHrP and PTH occurred after the amphibian divergence. The tetrapod PTH-L probably acquired a minor role and became potentially non-functional and was consequently eliminated from the human genome. In contrast, PTH which in teleosts and *Xenopus *appears to be a paracrine factor seems to have gained a more important functional role during evolution and became a major endocrine factor with a restricted production in a highly specialized structure, the parathyroid gland [34]. Future studies using more in depth functional studies and other organisms will be needed to support this hypothesis.

## Conclusions

The parathyroid hormone family contains 3 principal members, PTH, PTHrP and the recently identified PTH-L. In teleosts there are 5 genes which encode PTHrP (2), PTH (2) and PTH-L and in tetrapods there are 3 genes (PTHrP, PTH and PTH-L), the exception is placental mammals which have 2 genes and lack a PTH-L. Gene structure of PTH and PTH-L seems to be conserved throughout the vertebrates while PTHrP gene structure is divergent and it has acquired new exons and alternative promoters. The highly conserved *Xenopus *and chicken N-terminal (1-34) region of PTH, PTHrP and PTH-L has the capacity to stimulate calcium uptake across, respectively, the frog skin and chicken chorionallantois membranes, indicating a conserved role in calcium metabolism possibly via similar receptors. It is hypothesized that PTH family genes appeared at approximately the same time during the vertebrate radiation and evolved via gene duplication/deletion events. During evolution PTH-L was lost from the genome of eutherian mammals, while PTH, which has a paracrine distribution in lower vertebrates, became the product of a specific endocrine tissue, the parathyroid gland and gained an important role in calcium regulation. The PTHrP gene acquired during its evolution a disparate and more complex organization in vertebrates probably associated with its paracrine nature and pluripotent functions.

## Methods

### Animals and tissue collection

Adult *Xenopus laevis *were purchased from Xenopus Express (France) and maintained at 22°C. Adult chickens (*Gallus gallus*) were supplied by a local farm. Frogs and chickens were anesthetized with diethyl ether (Merck) and euthanized by double pithing and decapitation, respectively. Fertile white leghorn chicken eggs were obtained from Quinta da Freiria (Serpa, Portugal) and kept in humid conditions in an automatic incubator (Brinseca OCTAGON 40) at 37.5°C with gentle rotation. Tissues were collected, immediately frozen in liquid nitrogen and stored at -80°C.

All animal experiments were performed in accordance with Portuguese legislation under a "Group-1" licence from the Direcção-Geral de Veterinária, Ministério da Agricultura, do Desenvolvimento Rural e das Pescas, Portugal.

### In silico identification and validation of PTH-like transcripts

Putative PTH-like genes were identified in the amphibian (*Xenopus tropicalis) *and chicken (*Gallus gallus*) genome and EST databases by sequence similarity searches using human PTH (AAH96144.1) and PTHrP (AAA60221) and *Takifugu rubripes *PTHA (CAG26460.1), PTHB (CAG26461.1), PTHrPA (CAB94712.1), PTHrPB (CAG26459.2) and PTH-L (CAG26462.1) and the default settings of tBLASTn [57]. Amphibian and chicken genomes were accessed via Ensembl (http://www.ensembl.org), Xenbase (http://www.xenbase.org) and NCBI (http://www.ncbi.nlm.nih.gov/), respectively. EST sequences were retrieved from NCBI and BBSRC ChickEST databases (http://www.chick.manchester.ac.uk/). Isoforms of *Xenopus *and chicken transcripts were named according to the size of the deduced mature protein and the length of their 5'UTR region (A to D). Puffer fish (*Takifugu rubripes*) and human (*Homo sapiens*) genome assemblies available in Ensembl and the human dbEST NCBI database were also interrogated with *Xenopus *and chicken PTH family members to identify potential novel isoforms.

Validation of transcripts was done by specific PCR amplifications using *Xenopus *and chicken cDNAs as template, the primers listed in Table 2 and the number of thermocycles and annealing temperatures adjusted for each amplicon. *Xenopus *reactions were cycled 40 times with annealing temperatures of 59°C for PTH, 55°C for PTHrPA, 58°C for PTHrPB and 57°C for PTH-L. For chicken the annealing temperatures and cycles were, respectively, 53°C and 30 for PTH, 55°C and 35 for PTHrPA, 58°C and 35 for PTHrPB and 55°C and 40 for PTH-L. All PCR products were analysed on 1.5% agarose gel and sequenced to confirm their identity.

**Table 2:** Primer pairs (same prefix ending in fw or rv) used to amplify the Xenopus and chicken PTH family members.

	*Xenopus laevis*	Chicken
**PTH**	*PTHfw: aggagacgggctgtgagtgag$*	*PTHfw: atgacttctacaaaaaatctg$*
	*PTHrv: tcattggatgccaggcttta$*	*PTHrv: tggcttagttttaaagagta$*
	PTH2fw: tcagatgaagttacaggac*	PTHfw: gcataaccttggagagcatcg*
	PTH2rv: cttagtgctatgcctatg*	PTHrv: cctctgggtcctggcatc*
**PTHrP**	PTHrPfw: cagtatctccacgacaaagg*$	*PTHrP(1-139)fw: ctgagagcccagtcttgga$*
	*PTHrP(1-131)rv: ttacctgtaatctaattcttcca$*	*PTHrP(1-139)rv: gggtaacaatttcagtaact$*
	*PTHrP(1-144)rv: cgggtgccgctcatctgc$*	*PTHrP(1-141)5utrAfw: gaagggagtagcacctgggc$*
	PTHrPrv: tggtggcagggagtaag*	*PTHrP(1-141)5utrBfw: ggcacctgcttttaaaaccc$*
		*PTHrP(1-141)5utrCfw: gctaacagaggaactgcgac$*
		*PTHrP(1-141)5utrDfw: aggactgacccctcctttcc$*
		*PTHrP(1-141)rv: gatcccctctactgatcttcc$*
		PTHrP(1-139)fw: agcaaagcctggaaaacg*
		PTHrP(1-139rv: gtggaaaagatacagcagaattacc*
		PTHrP(1-141)5utrAfw: caggcttgcggtgaggcta*
		PTHrP(1-141)5utrArv: gcgaaactccactgctgaaag*
		PTHrP(1-141)5utrBfw: tgacccctcctttccttgc*
		PTHrP(1-141)5utrBrv: ggcacagaataactcagaagaaac*
		PTHrP(1-141)5utrCfw: cagaggaactgcgacgaacaac*
		PTHrP(1-141)5utrCrv: gcgaaactccactgctgaaag*
		PTHrP(1-141)5utrDfw: ggcacctgcttttaaaaccc*
		PTHrP(1-141)5utrDrv: aaggttttgatgaaagataggaatcc*
**PTH-L**	*PTH-Lfw: gagagatcagttgcagagg$*	*PTH-Lfw: gaacgacaagagaaggaaag$*
	*PTH-Lrv: tgaaggatcccgctccatt$*	*PTH-Lrv: ctgcttcatcgggtttga$*
	PTHLfw: ttgaagaaataaatcgccagag*	PTHLfw: gataaggcgagggcatttcaag*
	PTHLrv: atgctgctgattctttgctgt*	PTHLrv: cctgctgctggctgtgtg*
**r18S**	18s fw tgacggaagggcaccaccag*	18s rv aatcgctccaccaactaagaacgg*

Note: $ indicates primers for RT-PCR and * indicates primers for q-PCR. For *Xenopus *PTHrP the same forward primer was used for each pair.

### RNA extractions and quantitative gene expression

Total RNA (tRNA) extracted from adult frog and chicken tissues using Tri Reagent (Sigma Aldrich, Spain) was treated with 1 U DNase (DNA-free Kit, Ambion, UK) for 30 min at 37°C. DNase treated tRNA (500 ng) was denatured at 65°C for 5 min, quenched on ice for 5 min and used for cDNA synthesis in a 20 μl reaction volume containing 10 ng of pd(N)6 random hexamers (GE Healthcare, UK), 2 mM dNTPs, 100 U of MMLV-RT and 20 U RNasin^® ^Plus RNase inhibitor. cDNA was synthesized for 10 min at 20°C followed by 50 min at 42°C and 72°C for 5 min.

Quantitative real-time PCR (q-PCR) amplifications of *Xenopus *and chicken cDNAs used the primers listed in Table 2 designed with Primer Premier and Beacon Design software (Premier Biosoft Int., Palo Alto, CA). Triplicate reactions (20 μl final volume) containing 1 μl of template cDNA and 1 pmol of each primer were prepared and reactions repeated twice using Power SYBR Green PCR master mix (Applied Biosystems, Foster City, CA, USA) and a Bio-Rad iClycler iQ thermocycler system (software version 3.1.7050, Bio-rad, Life Science Group, USA). The thermocycle consisted of an initial step at 95°C for 10 min followed by 55 cycles of 95°C for 30 sec, 20 sec at an appropriate temperature for annealing of each primer pair, and 72°C for 30 sec. Annealing temperatures were 60°C for r18S; 56°C, 59°C and 53°C for the *Xenopus *PTH, PTHrP and PTH-L, respectively; and for the chicken amplicons 53°C for PTHrP(1-141)*5utrB*; 55°C for PTHrP(1-139); 57°C for PTH, PTH-L and PTHrP(1-141)*5utrC*; 58°C for PTHrP(1-141)*5utrD*; and 60°C for PTHrP(1-141)*5utrA*. Melting curves were performed to detect nonspecific products and primer dimers. PCR products were quantified relative to a standard curve constructed using serial dilutions of linearized DNA plasmid of the target transcript. Genomic contamination was monitored by including tRNA samples without MMLV-RT and r18S was used as the internal quantitative control for normalization. Relative gene expression was calculated as: number of copies (NC) = (A × 6.022 × 10^23^)/(B × 1 × 10^9 ^× 650 kDa), where A is the template quantity (ng of vector plus insert), B the template length (bp vector plus insert), and 650 kDa is the average weight of a base pair according to [58].

### Sequence comparisons and phylogenetic analysis

Multiple sequence alignments of PTH family prepro- and mature proteins were performed with ClustalX using the following parameters: Gonnet series matrix, Gap opening penalty 10, Gap extension 0.2 [59]. Alignments were displayed in GeneDoc (http://www.psc.edu/biomed/genedoc), manually edited and percentages of sequence identity and similarity calculated. Phylogenetic analysis was performed using both Neighbor Joining and Maximum Parsimony Methods [60] with 1000 and 100 bootstrap replicates, respectively, using MEGA 3.1 software [61].

### Gene organization and linkage analysis

The gene organization of the *Xenopus *and chicken PTH-like members was deduced using Spidey software (mRNA-to-genomic alignment; http://www.ncbi.nlm.nih.gov/IEB/Research/Ostell/Spidey) with the aid of Ensembl *in silico *gene annotation and the predicted structures were manually edited using intron/exon splice boundary consensus sequences (AG/GT) and by comparison with available EST data. The immediate gene environment of the PTH, PTHrP and PTH-L genes in *Xenopus *was assessed from scaffold annotation in Ensembl and the chicken homologue regions using Mapview (http://www.ncbi.nlm.nih.gov/mapview). To verify if conservation of the PTH-L genome region in amphibian and chicken genomes also exists in human and puffer fish, a search for homologues of neighbouring genes was performed.

### Electrophysiological measurements and unidirectional calcium fluxes

Previous studies established that PTH promotes calcium transport across the frog skin [52] and chicken chorionallantois membranes (CAMs) [27] thus providing assays to test the activity of the new PTH family peptides. The effect of *Xenopus *and chicken PTH(1-34), PTHrP(1-34) and PTH-L(1-34) (Genemed Synthesis, Inc., San Antonio, Texas, USA) on calcium transport was assessed *in vitro *using Ussing chambers with adult *Xenopus *abdominal skin and CAMs from chicken embryos of 16 to 18 days [stages 42 HH and 44 HH, respectively; [62]]. Human PTH (1-34) (Bachem, Germany) and salmon Luteinizing hormone-releasing hormone (LHRH, Bachem) were used as positive and negative controls, respectively.

The *Xenopus *experiments were carried out at 22-23°C in Ringers solution (2.4 mM NaHCO_3; _113.8 mM NaCl; 1.9 mM KC_2_H_3_O_2_; 1 mM CaCl_2_; 2.1 mM NaC_2_H_3_O_2_; 0.5 mM Mg(C_2_H_3_O_2_)_2 _and 5 mM glucose) at pH 8.1 with oxygenation provided by atmospheric air. The chicken experiments were carried out at 37°C using a standard bathing solution [130 mM NaCl; 1 mM MgSO_4_; 2 mM CaCl_2_; 8 mM KH_2_PO_4_; 15 mM glucose [63]] at pH 7.4 maintained by gassing with a mixture of 5% CO_2 _in O_2_.

The *Xenopus *and chicken membranes were pinned over the circular aperture of Ussing chambers (1 cm^2^) and 8 ml of saline solution was added to each half-chamber. Epithelial preparations were stabilized for 30 min and the saline solution was replaced before the addition of radioactive labelled ^45^Ca^2+ ^(0.75 μCi/ml CaCl_2_; GE Healthcare, UK) to the mucosa/chorion side. Time zero was established 15 min after ^45^Ca^2+ ^addition. Fluid samples (400 μl) were collected every 30 minutes over a total of two hours from the serosa/allantoic side and the first 2 samples served as controls prior to peptide (10 nM) addition. The volume sampled was replaced by an equal amount of saline and replicate 200 μl samples of the mucosa/chorion side were used to calculate calcium specific activity by counting in a liquid scintillation counter (Beckamn LS 6000IC, USA). All radiotracer experiments were performed under short circuit conditions.

Bio-electrical variables were recorded with a DVC-1000 voltage-clamp amplifier (WPI, Sarasota, US) by means of Ag-AgCl electrodes connected to the chamber by agar bridges (2 M KCl/3% agar) and data was collected via a Data-Trax acquisition system (WPI, Sarasota, US) connected to a personal computer (PC). At the start of experiments the trans-epithelial potential (Vt, mV) was recorded to check tissue integrity and then short circuited (Vt = 0) for subsequent experiments. Short circuit current (Isc, μAmp/cm^2^) was constantly recorded and the current deflections produced by 3 mV pulses once every minute were used for calculation of tissue resistance (*R*t, Ω.cm^2^) using Ohm's law.

Calcium fluxes were calculated using the equation: JinCa2+=Δ[C45a]BI(1/SAAP)(volumeBI)/[(time)(area)], where Δ[^45^*Ca*]_*BI *_represents the increase in radioactivity in the basolateral side (BI) half-chamber and SA_Ap _the apical side (Ap) specific activity (cpm/nmol) [56]. Data is presented as mean ± standard error of the mean (SEM). A paired Student's t-test was used to test the effect of peptide on calcium fluxes and one-way ANOVA was used to test the effect of peptide on electrophysiological measurements (Isc e Rt) using SigmaStat v.3.11 (Systat software, Inc., USA). The significance level was 5%.

## Authors' contributions

PLCP did the experimental work on chicken (database searches, cDNA isolation, PCR, membrane transport), analyzed results and wrote the initial draft of the manuscript; JCRC supervised the comparative and phylogenetic analysis and contributed to the writing; ASG did the experimental work on Xenopus (database searches, cDNA isolation, PCR, membrane transport) analysed results and contributed to the writing; JF supervised the membrane transport work and contributed to the writing; DMP contributed to the planning of the work, analysis of results and writing; AVMC devised the work, obtained funds, analysed results and contributed to the writing of the manuscript. All authors read and approved the final manuscript

## Supplementary Material

Additional file 1 ***Xenopus *and chicken parathyroid family gene and transcript data** Accession numbers (GenBank and Ensembl IDs) of nucleotide sequences, gene scaffolds and tissue of origin of EST of the vertebrate PTH family members. Click here for file

Additional file 2 **Multiple sequence alignment of vertebrate PTH family members**. The signal peptide (SP) is indicated by a double arrow and the 1-34 mature peptide is boxed. Potential proteolytic cleavage sites are in bold and italics and the Pre and Pro sites are indicated. The M-H-N amino acid motif is indicated in bold. Amino acid conservation is denoted by "*" and accession number of the sequences used are indicated in Figure 1. Click here for file

Additional file 3 **PTHrP amino acid multiple sequence alignment**. Description: The signal peptide (SP) is indicated by a double arrow and the three potential peptides (1-34PTHrP, mid-region and osteostatin) generated from the human precursor are indicated within boxes. The alignment includes various human, chicken and *Xenopus *PTHrP isoforms which are annotated according to the length of the mature protein sequence. Potential cleavage sites are in italics and bold and the Pre and Pro cleavage sites are indicated by arrows. The tetrapod L-H-D and the teleost M-H-D motifs are annotated in bold. The two lamprey PTH-like sequences were not included in the alignment since only the mature peptide region was characterized. Amino acid conservation is denoted by "*" and accession numbers of the sequences used are described in Figure 1. Click here for file

Additional file 4 **PTH-L amino acid multiple sequence alignment**. The signal peptide (SP) is indicated by a double arrow and the potential mature PTH-L peptide is within a box. The potential M-H-D motif previously identified in the *Takifugu *precursor is in bold and potential proteolytic cleavage sites within the signal peptide sequence are in bold and italics. Sequences underlined and in *italics *have been predicted *in silico *but were not confirmed by RT-PCR. Amino acid conservation is denoted by "*" and accession numbers are indicated in Figure 1. Click here for file

Additional file 5 **Chicken PTHrP alternative transcripts**. The five PTHrP transcripts that result from alternative exon skipping events are mapped against the structure of the chicken PTHrP gene and their respective EST accession number and size (bp) indicated. The novel chicken transcripts were named according to the size of the deduced mature peptide (139 or 141) precursor and the length of their 5'UTR region (A to D). Arrows delimit regions amplified by q-PCR for each transcript and the deduced mature peptide sequence of each transcript is given and +1 indicates the start of the mature peptide. Coding exons are represented by filled boxes, non-coding exons by open boxes and introns by lines and the dotted-filled boxes indicate the mature PTHrP peptide region. Non-coding 5'UTR exons are designated by E1' to E3' and the predicted intron sizes (bp) of the chicken PTHrP gene are given. For simplicity, the 5'UTR regions transcribed from non-coding exons are designated by letters (a to f) and the dashed/dotted line within the E2' and E1 region indicate the alternative splicing events. PTHrP 5'utrD EST was found to be incomplete and only part of the mature PTHrP peptide was characterized. Click here for file

Additional file 6 **RT-PCR expression profile of PTH/PTHrP family members in adults of *Xenopus *(A) and chicken (B)**. Gene specific primers were designed in order to amplify the *Xenopus *and chicken PTH and PTH-L transcripts and the PTHrP isoforms. In (A) the adult *Xenopus *tissues analyzed were spleen (1), skin (2), muscle (3), cartilage (4), bone (5), kidney (6), gall bladder (7), esophagus (8), stomach (9), duodenum (10), hindgut (11), midgut (12), liver (13), brain (14), lung (15), heart (16), gonads (17) and thyroid (18 and 19). In (B) the adult chicken tissues analyzed were forebrain (1), midbrain (2), hindbrain (3), pituitary (4), parathyroid (5), thyroid (6), bone (7), cartilage (8), muscle (9), kidney (10), liver (11), lung (12), eggs (13), duodenum (14), hindgut (15) and midgut (16). C ^(-) ^represents the negative control reaction. The ribosomal unit 18S was used as an internal control to normalize RT-PCR reactions and amplified products were sequenced to confirm identity. Click here for file
