# Supplementary material for: Gene structure, transcripts and calciotropic effects of the PTH family of peptides in Xenopus and chicken
Source: BMC Evol Biol. 2010 Dec 1;10:373. doi: 10.1186/1471-2148-10-373 (PMC3009671; doi:10.1186/1471-2148-10-373)
Supplement: Additional file 1 — Xenopus and chicken parathyroid family gene and transcript data Accession numbers (GenBank and Ensembl IDs) of nucleotide sequences, gene scaffolds and tissue of origin of EST of the vertebrate PTH family members. [file 1471-2148-10-373-S1.PDF]

Supplementary Table 1 - Accession numbers (GenBank and Ensembl IDs) and scaffolds of the PTH family members in chicken and *Xenopus*.

	Xenopus			**Chicken**		
	**Gene**	**Transcripts**	**ESTs origin**	**Gene**	**Transcripts**	**ESTs origin**
**PTH**	Scaffold_235	FM955441*		NC 006088.2[+]	NM_205452[†]	
				ENSGALG00000017295	*CV890868*<br>*CV041147*	Mix tissues (brain, ultimobranchial and parathyroid glands, cecal tonsil, primordial germ cells)
**PTHrP**	ENSXETG00000001307	FM955442*		NC_006092.2[+]	NM_205338[†]	
		CR437266	tailbud (stage 28-30)	ENSGALG00000005358	*BU384898*	trunks (stage 36)
		CR433007	tailbud (stage 28-30)		*BU252785*	limbs (stage 36)
					BU252877	limbs (stage 36)
					BM489067	Muscle (breast and leg); epiphyseal growth plate
					ENSGALESTT00000030972	
**PTH-L**	Scaffold_169	*AL775245*	gastrula (stages 10.5- 12)	Contig 68.24[+]	FM955443*	
		AL964863	gastrula (stages 10.5- 12)	NW_001471609		
		AL965929	gastrula (stages 10.5- 12)			
		BX750389	gastrula (stages 10.5- 12)			
		BX764109	gastrula (stages 10.5- 12)			
		CN076482	brain and spinal cord			
		CN076481	(tadpoles stage 58-64)			

The accession number of the predicted genes is indicated or when not available their putative location in genome regions in ENSEMBL assembly (*Xenopus* Scaffold_235 and Scaffold_169; chicken Contig 68.24) or NCBI assembly if available (chicken NC 006088.2 and NC_006092.2 indicated by a [+]). Accession numbers for ESTs identified in this study are indicated in italics and highlighted with an [*] are the GeneBank accession numbers of isolated cDNAs.
