# Supplementary material for: Gene structure, transcripts and calciotropic effects of the PTH family of peptides in Xenopus and chicken
Source: BMC Evol Biol. 2010 Dec 1;10:373. doi: 10.1186/1471-2148-10-373 (PMC3009671; doi:10.1186/1471-2148-10-373)
Supplement: Additional file 2 — Multiple sequence alignment of vertebrate PTH family members. The signal peptide (SP) is indicated by a double arrow and the 1-34 mature peptide is boxed. Potential proteolytic cleavage sites are in bold and italics and the Pre and Pro sites are indicated. The M-H-N amino acid motif is indicated in bold. Amino acid conservation is denoted by "*" and accession number of the sequences used are indicated in Figure 1. [file 1471-2148-10-373-S2.PDF]

```
                              SP
Chicken   : MFLPQRPLQMVTFLAIFFLACFATRQSTENRRAVTEHQLMHDKARAFQGLKRLLWLHNALGSVHTASSRDISLSNA :  76
Xenopus   : MFLSQKYLQIVTFIGIMCFACLAASENTQNERSVAEAQLMHDKGKTIEEISRQRWLQGLLGSVHNPGRRNVPLIKN :  76
Zebrafish : MLKMQRSQQRVALMMLMVVAAVHCQE-SESRRAVTEHQLMHDRGRSIQSLKRLIWLSSAIEGLHTAQARTLEPDSR :  75
Takifugu  : MQAYHGCAQRLA-IVLLVLTSCRGQE-DESRRSVTEHQLMHDRGRNIQSLKRLFWLSSAIEGLHTAQSRSAALNP- :  73
            *   ..  * *  * *. ..     * .* .* *.*.******.* .* *.*. **  .*  **..  *   .

Chicken   : IWDSQKSRDPSDLYNSISRDETSNPMKQLLELIEDEQGFPLLRKQYLLQNPKTPEGNWNPQDVFDLLQIKEFGRRR : 152
Xenopus   : FR------------------------RDLRRLNEEQQRFSSIRN---IRN----------------LQYKQAILAH : 109
Zebrafish : WR-----------------------SRGAQLYSQPGREESSGGQKRALET----------------LLSDLYRAHL : 112
Fugu      : -------------------------VKVPNLAMNP----SLGSAQPAVKS----------------LLRGFFNPYL : 104
                                      *            .       *                  *    .

Chicken   : NHSNQPQNFSH : 163
Xenopus   : NKNLMERDP-- : 118
Zebrafish : TFGLGEPEK-- : 121
Fugu      : TQ-LPEPEA-- : 112
               . .
```

Supplementary Figure 1 - Multiple sequence alignment of vertebrate PTH family members. The signal peptide (SP) is indicated by a double arrow and the 1-34 mature peptide is boxed. Potential proteolytic cleavage sites are in bold and italics and the Pre and Pro sites are indicated. The M-H-N amino acid motif is indicated in bold. Amino acid conservation is denoted by "*" and accession number of the sequences used are indicated in Figure 1.
