# Supplementary material for: Gene structure, transcripts and calciotropic effects of the PTH family of peptides in Xenopus and chicken
Source: BMC Evol Biol. 2010 Dec 1;10:373. doi: 10.1186/1471-2148-10-373 (PMC3009671; doi:10.1186/1471-2148-10-373)
Supplement: Additional file 3 — PTHrP amino acid multiple sequence alignment. Description: The signal peptide (SP) is indicated by a double arrow and the three potential peptides (1-34PTHrP, mid-region and osteostatin) generated from the human precursor are indicated within boxes. The alignment includes various human, chicken and Xenopus PTHrP isoforms which are annotated according to the length of the mature protein sequence. Potential cleavage sites are in italics and bold and the Pre and Pro cleavage sites are indicated by arrows. The tetrapod L-H-D and the teleost M-H-D motifs are annotated in bold. The two lamprey PTH-like sequences were not included in the alignment since only the mature peptide region was characterized. Amino acid conservation is denoted by "*" and accession numbers of the sequences used are described in Figure 1. [file 1471-2148-10-373-S3.PDF]

```
                                                     Pre        Pro
                                  SP
Human       : MIPAK---DMAKVMIVMLAICFLTKSDGKSVK-KRSVSEIQLMHNLGKHLNSMERVEWLRKKLQDVHNFVALGA :  70
Mouse       : MMSAN---TVAKVMIIMLAVCLLTQTDGKPVR-KRAVSEIQLMHNLGKHLASMERMQWLRRKLQDMHNFVSLGV :  70
Chicken     : MTSTK---NLAKAIVILYAICFFTNSDGRPMM-KRSVSEMQLMHNLGEHRHTVERQDWLQMKLQDVHSALEDAR :  70
Xenopus     : MNFLT---DLTKIGIILCGLSYLTECGCRPVK-RRAVSELQLMHNYGEFIHSSQRQEWLQMKLQDMYSASVSTP :  70
ZebrafishA  : MVSINGNLLKRVIFVTVFILLFSTNAESRPLRSKRAVNEVQLMHNLGVHKHVELRQDWLQMKLRGIHTASVKNT :  74
ZebrafishB  : MLLIR-CLEKAMLIIVLWSLCSFLFLEGLPIS-KRSISEVQLMHNVREHKEMLDRQDWLQLKLNNIIIPSVNDS :  72
TakifuguA   : MP----NLHWKTFFLSLCLLRLLALGEGHTLR-KRTVSEVQLMHNLGEHKQVQERREWLQRRLHGIHPIGLNND :  69
TakifuguB   : MFSLS-HFETLTLILFLSSLH----IEAKPLR-KRTISEVQLMHNVREHKQVGERQDWLQEKLKDVIVVSAKP- :  67
              *           . * *  *        ..*. ** *.*******.. .   .*  **. ** .*

Human       : PLAPRDA-----GSQRPRKKEDNVLVESHEKSLGEADKADVNVLTKAKSQ : 115
Mouse       : QMAARDG-----SHQKPTKKEENVLVDGNPKSLGEGDKADVDVLVKSKSQ : 115
Chicken     : TQRPRNKEDIVLGEIRNRRLLPEHLRAAVQKKSIDLDKAYMNVLFKTKP- : 119
Xenopus     : QESTPKIADHRPPKPQKHKMEENKPKTAPQKTLHRHSTKDNTLKPGIQ-- : 118
ZebrafishA  : D-------------------IIPELKELYPGEAEEIMAVLEKLMNPS--- : 102
ZebrafishB  : QKERKG-----------------KTNGPSVKRLRKGEGATWSFY------ :  99
TakifuguA   : DEPGWGR-------KGIASRELPNLRDLTPEEIQNALNVLEELLKSQE-- : 110
TakifuguB   : QHAHTG-----------------NIKSLFSNDVLRSN--MWKT------- :  91
                                      .       .         .
```

Supplementary Figure 2 - PTHrP amino acid multiple sequence alignment. The signal peptide (SP) is indicated by a double arrow and the three potential peptides (1-34PTHrP, mid-region and osteostatin) generated from the human precursor are indicated within boxes. The alignment includes various human, chicken and Xenopus PTHrP isoforms which are annotated according to the length of the mature protein sequence. Potential cleavage sites are in italics and bold and the Pre and Pro cleavage sites are indicated by arrows. The tetrapod L-H-D and the teleost M-H-D motifs are annotated in bold. The two lamprey PTH-like sequences were not included in the alignment since only the mature peptide region was characterized. Amino acid conservation is denoted by “*” and accession numbers of the sequences used are described in Figure 1.
