# Supplementary material for: Gene structure, transcripts and calciotropic effects of the PTH family of peptides in Xenopus and chicken
Source: BMC Evol Biol. 2010 Dec 1;10:373. doi: 10.1186/1471-2148-10-373 (PMC3009671; doi:10.1186/1471-2148-10-373)
Supplement: Additional file 5 — Chicken PTHrP alternative transcripts. The five PTHrP transcripts that result from alternative exon skipping events are mapped against the structure of the chicken PTHrP gene and their respective EST accession number and size (bp) indicated. The novel chicken transcripts were named according to the size of the deduced mature peptide (139 or 141) precursor and the length of their 5'UTR region (A to D). Arrows delimit regions amplified by q-PCR for each transcript and the deduced mature peptide sequence of each transcript is given and +1 indicates the start of the mature peptide. Coding exons are represented by filled boxes, non-coding exons by open boxes and introns by lines and the dotted-filled boxes indicate the mature PTHrP peptide region. Non-coding 5'UTR exons are designated by E1' to E3' and the predicted intron sizes (bp) of the chicken PTHrP gene are given. For simplicity, the 5'UTR regions transcribed from non-coding exons are designated by letters (a to f) and the dashed/dotted line within the E2' and E1 region indicate the alternative splicing events. PTHrP 5'utrD EST was found to be incomplete and only part of the mature PTHrP peptide was characterized. [file 1471-2148-10-373-S5.PDF]

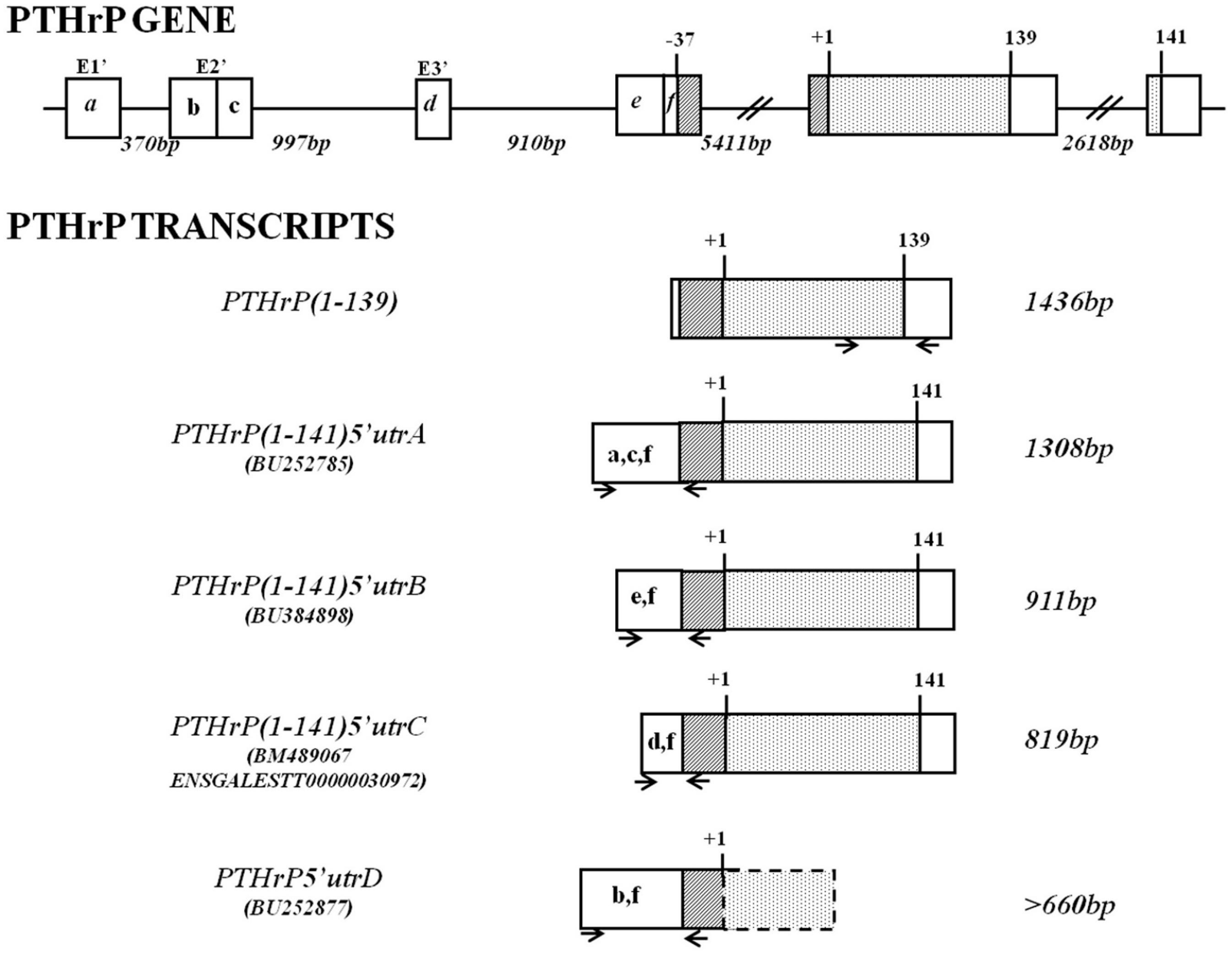

Supplementary Figure 4 - Chicken PTHrP alternative transcripts. The five PTHrP transcripts that result from alternative exon skipping events are mapped against the structure of the chicken PTHrP gene and their respective EST accession number and size (bp) indicated. The novel chicken transcripts were named according to the size of the deduced mature peptide (139 or 141) precursor and the length of their 5'UTR region (A to D). Arrows delimit regions amplified by q-PCR for each transcript and the deduced mature peptide sequence of each transcript is given and +1 indicates the start of the mature peptide. Coding exons are represented by filled boxes, non-coding exons by open boxes and introns by lines and the dotted-filled boxes indicate the mature PTHrP peptide region. Non-coding 5'UTR exons are designated by E1' to E3' and the predicted intron sizes (bp) of the chicken PTHrP gene is given. For simplicity, the 5'UTR regions transcribed from non-coding exons are designated by letters (a to f) and the dashed/dotted line within the E2' and E1 region indicate the alternative splicing events. PTHrP 5'utrD EST was found to be incomplete and only part of the mature PTHrP peptide was characterized.
