# Supplementary material for: Gene structure, transcripts and calciotropic effects of the PTH family of peptides in Xenopus and chicken
Source: BMC Evol Biol. 2010 Dec 1;10:373. doi: 10.1186/1471-2148-10-373 (PMC3009671; doi:10.1186/1471-2148-10-373)
Supplement: Additional file 6 — RT-PCR expression profile of PTH/PTHrP family members in adults of Xenopus (A) and chicken (B). Gene specific primers were designed in order to amplify the Xenopus and chicken PTH and PTH-L transcripts and the PTHrP isoforms. In (A) the adult Xenopus tissues analyzed were spleen (1), skin (2), muscle (3), cartilage (4), bone (5), kidney (6), gall bladder (7), esophagus (8), stomach (9), duodenum (10), hindgut (11), midgut (12), liver (13), brain (14), lung (15), heart (16), gonads (17) and thyroid (18 and 19). In (B) the adult chicken tissues analyzed were forebrain (1), midbrain (2), hindbrain (3), pituitary (4), parathyroid (5), thyroid (6), bone (7), cartilage (8), muscle (9), kidney (10), liver (11), lung (12), eggs (13), duodenum (14), hindgut (15) and midgut (16). C (-) represents the negative control reaction. The ribosomal unit 18S was used as an internal control to normalize RT-PCR reactions and amplified products were sequenced to confirm identity. [file 1471-2148-10-373-S6.PDF]

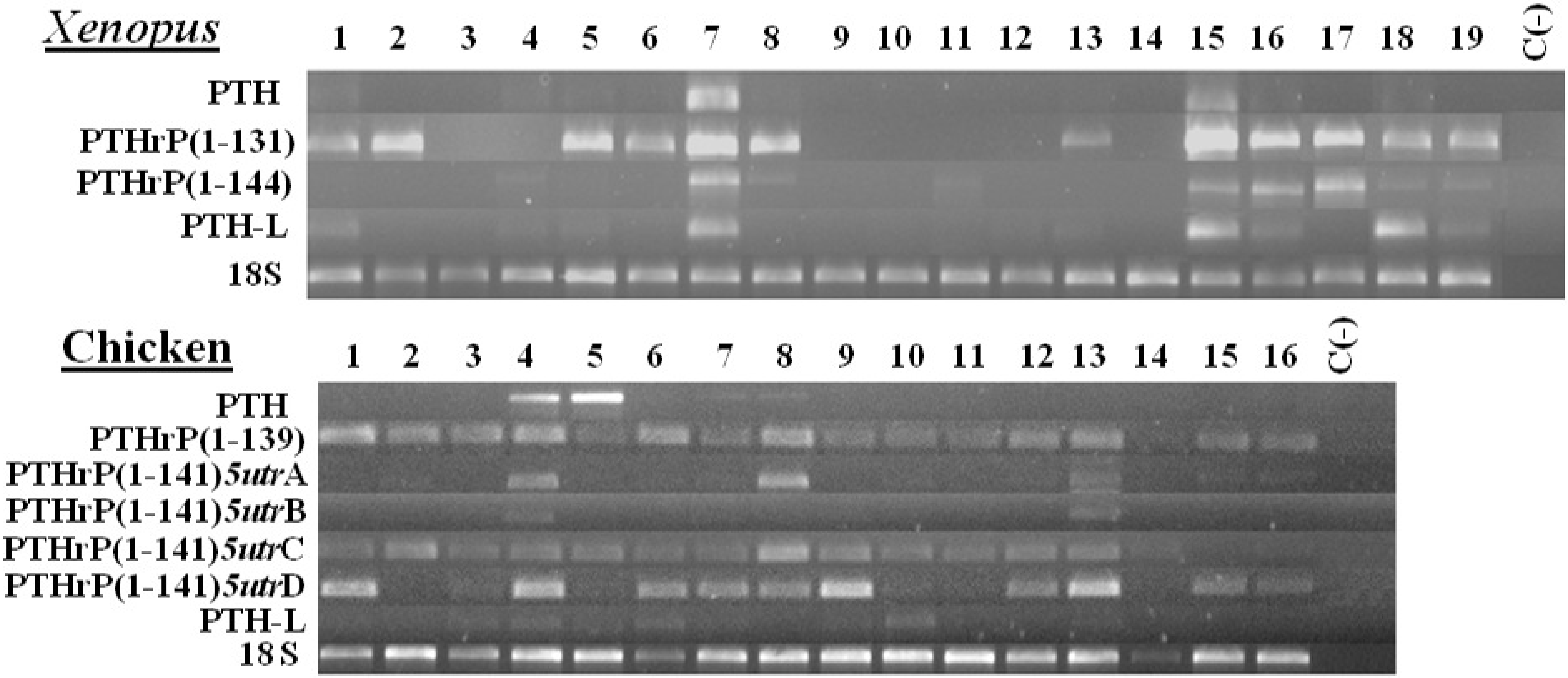

Supplementary Figure 5 - RT-PCR expression profile of PTH/PTHrP family members in adults of Xenopus (A) and chicken (B). Gene specific primers were designed in order to amplify the Xenopus and chicken PTH and PTH-L transcripts and the PTHrP isoforms. In (A) the adult Xenopus tissues analyzed were spleen (1), skin (2), muscle (3), cartilage (4), bone (5), kidney (6), gall bladder (7), esophagus (8), stomach (9), duodenum (10), hindgut (11), midgut (12), liver (13), brain (14), lung (15), heart (16), gonads (17) and thyroid (18 and 19). In (B) the adult chicken tissues analyzed were forebrain (1), midbrain (2), hindbrain (3), pituitary (4), parathyroid (5), thyroid (6), bone (7), cartilage (8), muscle (9), kidney (10), liver (11), lung (12), eggs (13), duodenum (14), hindgut (15) and midgut (16). C (-) represents the negative control reaction. The ribosomal unit 18S was used as an internal control to normalize RT-PCR reactions and amplified products were sequenced to confirm identity.
