# Supplementary material for: Gene structure, transcripts and calciotropic effects of the PTH family of peptides in Xenopus and chicken
Source: BMC Evol Biol. 2010 Dec 1;10:373. doi: 10.1186/1471-2148-10-373 (PMC3009671; doi:10.1186/1471-2148-10-373)
Supplement: Additional file 4 — PTH-L amino acid multiple sequence alignment. The signal peptide (SP) is indicated by a double arrow and the potential mature PTH-L peptide is within a box. The potential M-H-D motif previously identified in the Takifugu precursor is in bold and potential proteolytic cleavage sites within the signal peptide sequence are in bold and italics. Sequences underlined and in italics have been predicted in silico but were not confirmed by RT-PCR. Amino acid conservation is denoted by "*" and accession numbers are indicated in Figure 1. [file 1471-2148-10-373-S4.PDF]

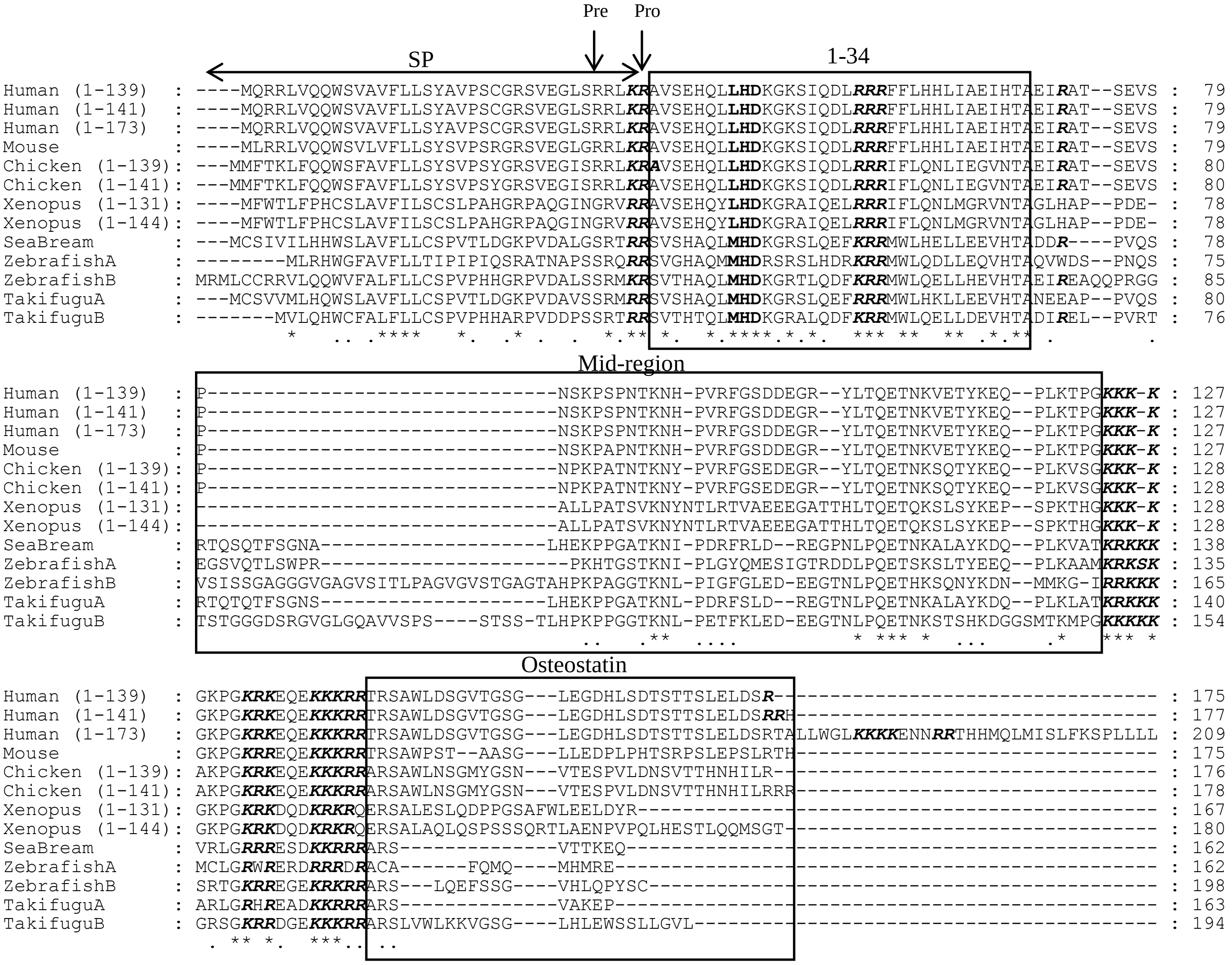

Supplementary Figure 3 - PTH-L amino acid multiple sequence alignment. The signal peptide (SP) is indicated by a double arrow and the potential mature PTH-L peptide is within a box. The potential M-H-D motif previously identified in the Takifugu precursor is in bold and potential proteolytic cleavage sites within the signal peptide sequence are in bold and italics. Sequences underlined and in italics have been predicted in silico but were not confirmed by RT-PCR. Amino acid conservation is denoted by "*" and accession numbers are indicated in Figure 1.
